# *Wolbachia* pseudogenes and low prevalence infections in tropical but not temperate Australian tephritid fruit flies: manifestations of lateral gene transfer and endosymbiont spillover?

**DOI:** 10.1186/s12862-015-0474-2

**Published:** 2015-09-18

**Authors:** Jennifer L. Morrow, Marianne Frommer, Jane E. Royer, Deborah C. A. Shearman, Markus Riegler

**Affiliations:** Hawkesbury Institute for the Environment, Western Sydney University, Locked Bag 1797, Penrith, NSW 2751 Australia; Evolution and Ecology Research Centre, School of Biological, Earth and Environmental Sciences, University of New South Wales, Sydney, NSW 2052 Australia; Queensland Department of Agriculture and Fisheries, EcoSciences Precinct, 41 Boggo Road, Dutton Park, QLD 4102 Australia

## Abstract

**Background:**

Maternally inherited *Wolbachia* bacteria infect many insect species. They can also be transferred horizontally into uninfected host lineages. A *Wolbachia* spillover from an infected source population must occur prior to the establishment of heritable infections, but this spillover may be transient. In a previous study of tephritid fruit fly species of tropical Australia we detected a high incidence of identical *Wolbachia* strains in several species as well as *Wolbachia* pseudogenes. Here, we have investigated this further by analysing field specimens of 24 species collected along a 3,000 km climate gradient of eastern Australia.

**Results:**

*Wolbachia* sequences were detected in individuals of nine of the 24 (37 %) species. Seven (29 %) species displayed four distinct *Wolbachia* strains based on characterisation of full multi locus sequencing (MLST) profiles; the strains occurred as single and double infections in a small number of individuals (2–17 %). For the two remaining species all individuals had incomplete MLST profiles and *Wolbachia* pseudogenes that may be indicative of lateral gene transfer into host genomes. The detection of *Wolbachia* was restricted to northern Australia, including in five species that only occur in the tropics. Within the more widely distributed *Bactrocera tryoni* and *Bactrocera neohumeralis*, *Wolbachia* also only occurred in the north, and was not linked to any particular mitochondrial haplotypes.

**Conclusions:**

The presence of *Wolbachia* pseudogenes at high prevalence in two species in absence of complete MLST profiles may represent footprints of historic infections that have been lost. The detection of identical low prevalence strains in a small number of individuals of seven species may question their role as reproductive manipulator and their vertical inheritance. Instead, the findings may be indicative of transient infections that result from spillover events from a yet unknown source. These spillover events appear to be restricted to northern Australia, without proliferation in host lineages further south. Our study highlights that tropical fruit fly communities contain *Wolbachia* pseudogenes and may be exposed to frequent horizontal *Wolbachia* transfer. It also emphasises that global estimates of *Wolbachia* frequencies may need to consider lateral gene transfer and *Wolbachia* spillover that may be regionally restricted, transient and not inherited.

**Electronic supplementary material:**

The online version of this article (doi:10.1186/s12862-015-0474-2) contains supplementary material, which is available to authorized users.

## Background

*Wolbachia pipientis* (Alphaproteobacteria) is a common endosymbiotic bacterium found in 40 to 50 % of insect, arachnid and terrestrial isopod species [1, 2]. In many host species *Wolbachia* is a reproductive parasite, while other host species may benefit from infections [3]. *Wolbachia* is mostly maternally inherited, but occasional horizontal transmission into uninfected lineages contributes to the large number of infected species [4–6]. Such switches of host lineages may result from hybridisation and introgression between closely related species [7]; sharing of ecological niches [8]; or transfer by or from parasitoids and predators [9]. The initial contact between *Wolbachia* of an infected with an uninfected host is the first essential step for the establishment of new *Wolbachia*-host associations. It must be followed by the colonisation of the host ovaries as a requirement for inheritance, as well as the induction of a reproductive manipulation or, alternatively, a positive fitness effect to promote its spread through new host populations [10, 11]. From an epidemiological perspective, this first exposure may be seen as a spillover of infectious agents from an infected source species to a new host species, and it may occur with or without its propagation in its new host species [12]. For *Wolbachia*, a model system for microbial symbiosis research, this initial spillover has so far not been differentiated from the more general concept of horizontal transfer itself. Most studies dealing with this phenomenon investigated horizontal transmission as the establishment of new heritable infections in new host lineages that may then express a reproductive phenotype leading to increases in infection frequencies in populations. However, not every spillover will result in the establishment of an inherited infection. Spillovers may still be detectable as non-heritable somatic and transient *Wolbachia* infections, or *Wolbachia* DNA [13] e.g. in digestive systems. It can be expected that the frequency of spillover is larger than the actual establishment of newly inherited infections, and this difference may inflate global estimates of actual infection frequencies. Another overestimation of infection frequencies (in particular in studies that rely on a single or a few marker genes) may stem from lateral transfer of *Wolbachia* genes into host genomes that can be seen as foot-prints of historic infections [14] that may occur without the presence of current infections, yet this has so far rarely been addressed in *Wolbachia* surveys [15].

*Wolbachia* spillover and with it *Wolbachia* horizontal transmission may be driven by ecological interactions between infected and uninfected host species, and may therefore be specific to habitat and climate. The diversity of ecological interactions is higher in tropical than temperate regions due to the increased biodiversity in the tropics [16, 17]. Within this existing conceptual framework it can be argued that *Wolbachia* spillover and horizontal transfer from infected to uninfected species should be more frequently observed in tropical than in temperate insect communities. However, this is not reflected in studies comparing the overall incidence of *Wolbachia*-infected species in arthropod communities from tropical and temperate regions that indicated parity of *Wolbachia* incidence across climatic zones [18–21]. On a different scale, *Wolbachia* prevalence within individual host species and populations was shown to vary from very low [13, 15, 22] to fixation [23, 24] but little information is available on how this varying infection prevalence within species is distributed across extensive latitudinal clines and different climatic regions, except for the studies on the *Wolbachia* prevalence in *Drosophila melanogaster* and *Drosophila simulans* populations along the east coast of Australia [25–27].

We have previously investigated *Wolbachia* horizontal transfer between host species and potential lateral gene transfer in Australian tephritid species by analysing fruit fly communities of tropical Australia [28]. True fruit flies of the family Tephritidae encompass approximately 5,000 species worldwide, including key pests of the genera *Anastrepha*, *Bactrocera, Ceratitis*, *Dacus* and *Rhagoletis* [29]. The largest proportion of Australian tephritids belongs to the genus *Bactrocera* with 97 endemic species [30, 31]. The majority originate from and are restricted to the tropical regions [32, 33]. However, since establishment of horticultural production in Australia in the 19^th^ century, several fruit fly species have expanded into more temperate regions, in particular due to invasive expansion and planting of host plants [34].

Double infections of two *Wolbachia* strains were found in individuals of six Australian tephritid fruit fly species of the genera *Bactrocera* and *Dacus* [28]. These two *Wolbachia* strains were characterised with two distinct and complete multi locus sequence typing (MLST) profiles, ST-285 and ST-289. Individuals of a seventh fruit fly species also carried ST-285 as a single infection. Two more *Wolbachia* strains, ST-17 and ST-370, also characterised with full sets of MLST genes, were detected in one individual of these *Bactrocera* species, *Bactrocera frauenfeldi*, and both of these strains were also found in individuals of a parasitoid wasp species, *Fopius arisanus* (Braconidae), that is commonly found parasitising Australian fruit fly larvae (including *B. frauenfeldi*). All infected tephritid species were sampled from tropical northern Queensland where they have overlapping plant host range [32], food sources [35] and parasitoid complexes [36], perhaps providing more opportunities for *Wolbachia* spillover and horizontal transfer into new lineages within that environment than further south. This earlier MLST based study also revealed the presence of pseudogenised MLST genes in two species, *Bactrocera peninsularis* and *Bactrocera perkinsi* [28].

Here we have expanded on our previous work by surveying more fruit fly individuals (including from additional species) collected over a significantly greater area of eastern Australia, from tropical to temperate regions and over a distance of about 3,000 km, with a focus on the two widespread and economically-relevant species *Bactrocera tryoni* and *Bactrocera neohumeralis*. The main objective of our study was to assess incidence and prevalence of the four previously characterised *Wolbachia* strains in Australian tephritid fruit flies from geographically distant populations in order to test whether these high levels of shared *Wolbachia* strains found in the tropics can also be seen in other regions of Australia. We hypothesised that, due to increased species diversity and interactions, the detection of *Wolbachia*, including the detection of pseudogenes, may be higher in the more tropical regions of Australia. We also hypothesised that an overall low prevalence of identical *Wolbachia* strains in individuals of several species in the tropics without proliferation in other regions may be a manifestation of a *Wolbachia* spillover effect while the detection of *Wolbachia* pseudogenes and failed amplification of other marker genes in all specimens of a species may suggest lateral transfer of *Wolbachia* genes into the fly genome and the loss of infections in such species.

## Methods

### Insect samples

This study included field collected and laboratory specimens of fruit flies belonging to the genera *Bactrocera*, *Dacus*, *Dirioxa* and *Ceratitis*. A total of 592 flies from 24 species were field-collected in New South Wales, Queensland, the Northern Territory and the Torres Strait Islands during two sampling periods, from 1996 to 2001, and from 2012 to 2013 (Table 1 and Additional file 1). While the majority of tephritid species reside in the equatorial and tropical regions of Australia, ten of the 24 species included in this study also occur in the subtropical and temperate regions [32]. Most flies were male and collected in the summer by trapping with male attractants cue lure [37], methyl eugenol for *Bactrocera visenda* and zingerone for *Bactrocera jarvisi* [33]. Both sexes of *B. tryoni*, *Bactrocera cacuminata* and *Dirioxa pornia* were collected directly on or from infested fruit. Fly specimens were identified under a stereomicroscope using identification keys [30]. Samples were selected based on availability and to canvas a range of species and populations. Prior to 2010, *Bactrocera aquilonis* was recognised as a distinct species that is morphologically similar to *B. tryoni* but found only in the Northern Territory and Western Australia [38]. Hybridisation between these two species is considered to have occurred in the Northern Territory [32] and it has been proposed that this species be synonymised with *B. tryoni* [39]; therefore, here all flies originally classified as *B. aquilonis* collected from the Northern Territory have been listed as *B. tryoni*. The species *Bactrocera papayae* (sampled from the Torres Strait Islands in 1998) has also recently been synonymised with *Bactrocera dorsalis* [40]. A subset of the fly samples (*n* = 104, mostly collected in equatorial and tropical Australia) were selected for MLST analysis and reported in Morrow *et al.* [28]. In addition to field-caught flies, we also screened eight females from each of the following laboratory lines: *B. tryoni*, *B. neohumeralis*, *B. jarvisi*, and *B. cacuminata* kept at Western Sydney University, Richmond, New South Wales; two independent *B. neohumeralis* lines from Cairns, Queensland, and *Ceratitis capitata* (Vienna 7/Mix 99) from Perth, Western Australia (Additional file 2) [41].

**Table 1:** *Wolbachia* prevalence in Australian fruit flies

		Total *Wolbachia* prevalence					
Fruit Flies	Abbreviation	No. individuals	No. *wsp* positive	No. 16S rDNA positive	No. with complete MLST	Prevalence (%)	MLST ST [28]
*Bactrocera allwoodi*	*Ba*	1	0	0	0	0	
*Bactrocera bryoniae*	*Bb*	51	4	3	4	7.8	285, 289
*Bactrocera cacuminata*	*Bca*	21	0	0	0	0	
*Bactrocera chorista*	*Bch*	10	0	0	0	0	
*Bactrocera decurtans*	*Bde*	6	1	1	1	16.7	285, 289
*Bactrocera dorsalis*	*Bdo*	1	0	0	0	0	
*Bactrocera fallacis*	*Bfa*	9	0	0	0	0	
*Bactrocera frauenfeldi*	*Bfr*	34	5	4	5	14.7	285, 289, 17, 370
*Bactrocera jarvisi*	*Bj*	10	0	0	0	0	
*Bactrocera manskii*	*Bma*	7	0	0	0	0	
*Bactrocera murrayi*	*Bmu*	3	0	0	0	0	
*Bactrocera neohumeralis*	*Bn*	132	13	12	13	9.8	285, 289
*Bactrocera quadrata*	*Bq*	9	0	0	0	0	
*Bactrocera strigifinis*	*Bs*	37	5	4	5	13.5	285, 289
*Bactrocera tryoni*	*Bt*	190	4	4	4	2.1	285
*Bactrocera visenda*	*Bv*	6	0	0	0	0	
*Dacus absonifacies*	*Dab*	6	0	0	0	0	
*Dacus aequalis*	*Dae*	13	0	0	0	0	
*Dacus axanus*	*Dax*	10	1	1	1	10	285, 289
*Dacus bellulus*	*Dbe*	3	0	0	0	0	
*Dacus newmani*	*Dn*	9	0	0	0	0	
*Dirioxa pornia*	*Dip*	12	0	0	0	0	
Pseudogenes^a^							
*Bactrocera peninsularis*	*Bpen*	7	0	7	0 (*fbpA* only)	0	n.a.
*Bactrocera perkinsi*	*Bper*	5	5	5	0 (all but *ftsZ*)	0	n.a.
Total		592	38	41	33		

^a^ *Bactrocera peninsularis* amplified strongly for 16S rDNA and *fbpA* but not for *wsp*, and the loci are therefore possibly pseudogenes. *Bactrocera perkinsi* amplified both 16S rDNA and *wsp*, however co-amplified pseudogenes for *wsp*, *coxA*, *hcpA* and *fbpA,* but lacked *ftsZ* [27]. Neither species was considered to harbour a true *Wolbachia* infection for any analyses

### DNA extraction, PCR, cloning and sequencing

Genomic DNA was extracted from individual fruit fly abdomens, containing the reproductive organs, while the remainder of the specimen was stored in ethanol at −80 °C for subsequent independent confirmation of positive results. Prior to DNA extraction, specimens were treated with 4 % sodium hypochlorite (Sigma, St Louis, MO) for 5 min, then triton-X (0.02 %) and then thoroughly rinsed with Milli-Q water to reduce surface contamination. DNA from *D. melanogaster* line *w*1118, infected with *Wolbachia* strain *w*MelPop [42], was used as a positive control. Insect tissue was ground in 1.5 mL microcentrifuge tubes with microtube pestles (Scientific Specialities Inc., Lodi, CA) and cell lysis performed overnight followed by extraction according to the GenElute Mammalian Genomic DNA Miniprep kit (Sigma) protocol. Elution of DNA from spin columns was performed with 100 μL nuclease-free water. Risk of contamination was minimised by routinely replacing stock solutions, dispensing aliquots of stock reagents and using filter tips for all DNA extractions and PCRs. Although cross contamination of flies caught in the same trap or stored in the same tube of ethanol after collection has previously been shown to be unlikely [43], we have further minimised this risk by surface treatment of samples with sodium hypochlorite prior to DNA extraction (as outlined above), plus independent extraction and PCR experiments in different laboratories.

PCR-based screening of fruit fly DNA (Additional file 3) was undertaken using the *Wolbachia* surface protein (*wsp*) and 16S rDNA loci. Primers for *wsp* were 81F and 691R [44] and Wsp-F and Wsp-R [45]. 16S rDNA was amplified with wspecF and wspecR [20]. Host mitochondrial *cytochrome c oxidase subunit I* (*COI*) fragments were amplified with Dick and Pat [46] as DNA quality control. Veracity of PCR results was tested by inclusion of no-template controls. All positive amplicons were confirmed by replication and further screening with other primer sets [28]. *COI* and *wsp* amplicons were prepared for direct sequencing by treatment with a combination of 0.5u Exonuclease I (New England Biolabs, Ipswich, MA) and 0.25u Shrimp Alkaline Phosphatase (Promega), with incubation at 37 °C for 30 min, then 95 °C for 5 min, prior to sequencing by Macrogen (Seoul, Korea).

For products displaying multiple sequences through direct sequencing, *wsp* was PCR amplified for cloning. Amplicons were either gel-extracted using the Wizard SV Gel and PCR Clean-up System (Promega) and eluted in 25 μL nuclease-free water; or used directly in the ligation reaction. Ligation was with 0.5 μL pGEM-T Easy vector (Promega), 1X Rapid ligation buffer and 3u T4 DNA ligase (Promega). Transformation of JM109 competent cells (Promega) was performed according to the manufacturer’s protocol. Colonies were smeared into a PCR tube using a sterile pipette tip and subjected to PCR using standard T7 Promoter and SP6 primers with reaction and cycling conditions as described for insect *COI* (Additional file 3). Positive clones, recognised by appropriately sized PCR products, were prepared for direct sequencing as described above. A minimum of three clones, but usually eight clones for each transformed ligate were selected for sequencing in both directions, using T7 Promoter and SP6 primers.

### DIG Southern hybridisation

PCR amplicons were also authenticated by Southern hybridisation using DIG DNA Labelling and Detection Kit (Roche Applied Sciences, Indianapolis, IN) based on the higher sensitivity method outlined in Arthofer *et al.* [47]. The *wsp* probe was generated as described in Additional file 3, using *D. melanogaster w*1118 DNA as template. This method was applied to all individuals, to also confirm the absence of *wsp* amplicons. Flies were classified as uninfected when repeated amplification attempts with *wsp* and 16S rDNA were negative but *COI* was positive. Individuals were considered *Wolbachia* infected when *wsp* and 16S rDNA primers amplified appropriately sized fragments from species for which we had established complete MLST profiles; *wsp* amplicons hybridised to the *wsp* probe; and *wsp* amplicons produced sequence homologues. The *COI* locus of selected infected and uninfected flies was sequenced to determine the host-mitochondrial association of *Wolbachia* sequences.

### PCR-RFLP

Single restriction enzyme digestion was performed on *wsp* amplicons to test for multiple infections and to confirm the presence of no more than the two sequence types revealed via clone sequencing. The sequence differences within two of the *wsp* alleles found as multiple infections enabled *Taq*I (cuts *wsp* allele 661 at position 516) and *Spe*I (cuts *wsp* allele 11 at position 286) to distinguish the alleles. *Taq*I and *Spe*I (Promega) reactions were performed according to the manufacturer’s protocols for 3 h. The samples were electrophoresed on 1.2 % agarose gels. Uncut, *Taq*I cut and *Spe*I uncut bands were independently excised from the gel. Samples were purified using the Wizard SV Gel and PCR Clean-up System and sequenced.

### Analyses of *Wolbachia* incidence and prevalence

*Wolbachia* incidence was defined as the percentage of species with individuals that were infected with a fully characterised *Wolbachia* strain (MLST genes, *wsp*, 16S rDNA). *Wolbachia* prevalence was defined as the percentage of infected individuals within a species [1]. Furthermore, species for which all individuals had *Wolbachia* pseudogenes and incomplete marker gene sets were considered as uninfected. While we sampled an average of 24 individuals per species, sample size was limited for some species. Thus, we restricted inferences about *Wolbachia* incidence to species for which we had at least ten individuals from within the same collection period. This was to reduce the risk of underestimating *Wolbachia* incidences in species where *Wolbachia* occurs at low prevalence and was comparable to other recent studies of infection frequencies [1, 48].

**Fig. 1:**
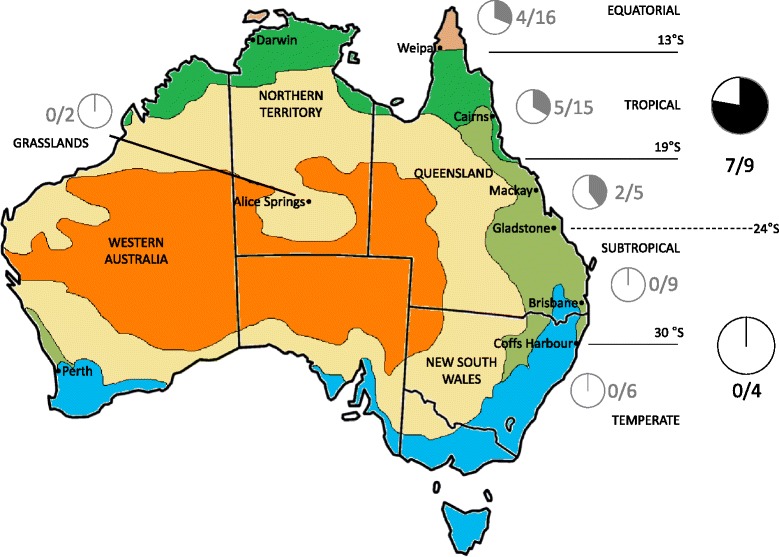
Incidence of *Wolbachia* infection in tephritid species. The Köppen map of Australia represents the climate groups and latitudes as approximate climate divisions along the east coast of Australia. *Wolbachia* incidence is presented as filled sections of pie graphs, with the number of infected species per total number of tested species within each of the six regions. Grey pie graphs represent *Wolbachia* incidence in all studied species of individual regions. Black pie graphs represent *Wolbachia* incidence in species for which at least ten individuals were available north or south of 24° latitude (set as midpoint for the analysis). *Wolbachia* incidence was higher in the northern half than the southern half of the latitudinal gradient (Fisher’s exact test; *p* < 0.05)

For analysis of incidence, sampling locations were placed into five climate groups as defined by the Australian Bureau of Meteorology, based on their shared Köppen climate classifications: equatorial, tropical, subtropical, temperate and grasslands (Fig. 1). The latitudinal gradient was then split in half, with the midpoint set at 24°, south of Gladstone, Queensland. *Wolbachia* incidence between the northern and southern halves of the gradient was then tested through Fisher’s exact test on the numbers of species with infected individuals versus species without infected individuals.

Prevalence was determined for twelve species only; species were selected based on either *Wolbachia* infection or availability of a minimum of 10 sampled individuals if uninfected. To incorporate the large differences in species distributions, a linear model (lm) was fitted to the relationship of *Wolbachia* prevalence with individual species distribution (Additional file 4), based on the midpoint latitude of each species’ geographic range as per Hancock *et al.* [32] and Royer and Hancock [33].

Prevalence of *Wolbachia* across geographic regions was only assessed for two widespread species, *B. tryoni* (*n* = 190) and *B. neohumeralis* (*n* = 132), as they were sampled from equatorial to temperate regions and had evidence of *Wolbachia* infections. The five Köppen climate groups were further divided into 13 latitudinal zones based on common sampling locations (Table 2). Infection prevalence across latitude and species was tested using multivariate generalised linear models (manyglm, negative binomial response) available in the package *mvabund* specifically designed for multivariate abundance data in community ecology [49] and this was performed in R 2.15 [50].

**Table 2:** *Wolbachia* prevalence across climate zones in two widely-distributed Australian fruit fly species

		Total *Wolbachia* prevalence			*Wolbachia* prevalence per climate zone (infected/total)												
					Equatorial		Tropical			Subtropical					Temperate		Grasslands
Fruit Flies	Abbreviation	No.	+ve	%	Torres Strait	Weipa/Coen	Darwin	Cairns	Townsville	Mackay	Gladstone	Bundaberg	Brisbane	Lismore	Coffs Harbour	Richmond	Alice Springs
					10–11°S	12–13°S	10–12°S	16–17°S	18–19°S	20–21°S	22–23°S	24–25°S	26–27°S	28–29°S	30–31°S	32–34°S	30–31°S
*B. neohumeralis*	*Bn*	132	13	9.8	0/11	n.d		9/37	0/12	3/12	1/10	0/10	0/28	0/10	0/2		
* B. tryoni*	*Bt*	190	4	2.1	n.d	0/13	0/6	2/40	0/10	2/12	0/12	0/10	0/37	n.d	0/11	0/19	0/20
Total		322	17		0/11	0/13	0/6	11/77	0/22	5/24	1/22	0/20	0/65	0/10	0/13	0/19	0/20

The latitudinal gradient of eastern Australia was divided into regions and represented by a major town or city. Darwin and Alice Springs are included as separate areas because they are not along the east coast of Australia. Regions are classified into climate groups according to the Köppen classification (Australian Bureau of Meteorology). *Bactrocera neohumeralis* does not occur in Darwin, Richmond or Alice Springs [32], whereas non-determined (n.d.) regions were not sampled

Fisher’s exact test was also applied to test *Wolbachia* prevalence over time within five species that were polymorphic for *Wolbachia* infections and were collected across the two sampling periods.

### Phylogenetic analyses

Phylogenetic analyses were performed for *COI* and *wsp* sequences in order to verify that mitochondrial lineages had independently acquired *Wolbachia* strains through horizontal transfer (and not through potential hybridisation with other species). It is also noted that phylogenetic analyses were not undertaken to infer species relationships, as single gene approaches are insufficient for such questions. DNA sequences were trimmed and edited in Sequencher 4.0 (GeneCodes Corporation) and then analysed in MEGA 5.05 [51]. *COI* and *wsp* genes were independently aligned (MUSCLE algorithm). Pairwise distance matrices were calculated for *COI* using number of differences and p-distance models. Substitution models were selected using Find Best DNA Model (ML), which calculated the lowest Bayesian Information Criterion score (GTR + G for *wsp*; TN93 + G for *COI*). Bayesian Inference phylogenies were produced by MrBayes 3.2 [52] running 10^7^ generations with a sample frequency of 100. The first 25 % of trees were discarded, and a 50 % majority rule consensus tree returned.

## Results

*Wolbachia*-specific primers for *wsp* and 16S rDNA were used to screen 592 field-collected Australian fruit flies representing 24 species of *Bactrocera*, *Dacus* and *Dirioxa.* Overall, individuals of eight species were positive for both *wsp* and 16S rDNA, while all individuals of one species, *B. peninsularis*, were positive for 16S rDNA without amplification of *wsp* (Table 1). All individuals from the independently established laboratory lines were negative for *Wolbachia* (Additional file 2). Initial screening using *wsp* primers 81F and 691R [44] appeared to produce false positives for some flies. Thus, primers Wsp-F and Wsp-R [45] were chosen, as their amplicons were more consistent and only occasionally produced spurious bands. Southern hybridisation with *wsp* was applied to all 592 samples, including those that initially appeared to be negative for *Wolbachia*; this confirmed specificity as well as improved detection sensitivity. In this way, four additional individuals, one each from four species (*B. bryoniae*, *B. frauenfeldi*, *B. neohumeralis* and *B. strigifinis*) with very faint *wsp* PCR amplification and undetectable 16S rDNA fragments using standard PCR were confirmed to carry *wsp* DNA by Southern hybridisation, while other individuals in these species were positive for both loci after standard PCR. We considered these four individuals infected with low titre *Wolbachia* because we had previously characterised complete MLST profiles in other individuals of these host species. Furthermore, control Southern hybridisation to samples with high titre infections demonstrated that the DIG-labelled *wsp* probe did not bind to primer-dimers or spurious *wsp* products, but hybridised to *wsp* amplicons that were verified by sequencing.

### Sequence analysis of *wsp* and 16S rDNA

**Fig. 2:**
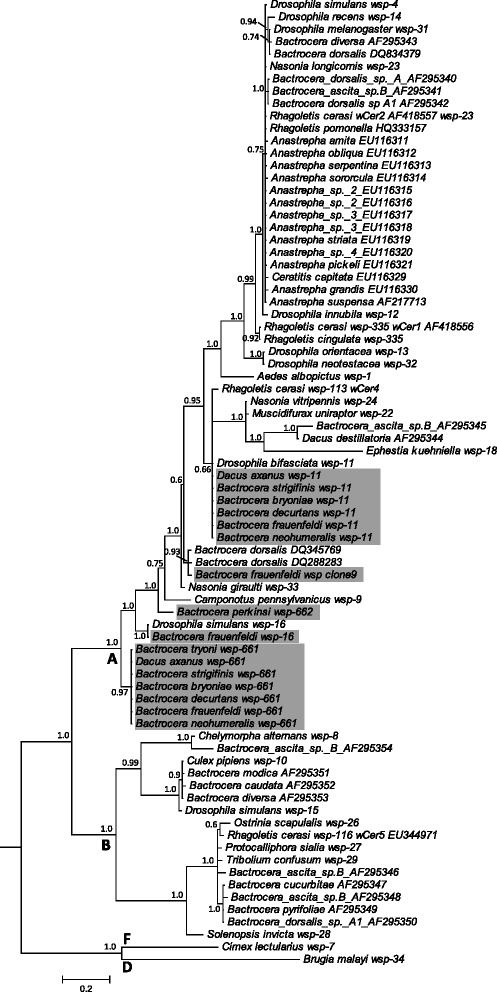
Bayesian inference tree of *wsp* sequences from *Wolbachia* within tephritid fruit flies and other invertebrates. Highlighted sequences are from this study (details in Table 3). All other sequences were retrieved from GenBank or the *Wolbachia wsp* database (accession number or *wsp* allele number following host species name). Supergroup classification of *wsp* sequences is marked at the nodes. Posterior probabilities >0.50 are marked at the nodes; tree was rooted by supergroup D and F strains. Scale bar represents the number of nucleotide substitutions per site

Thirty-eight flies amplified at the *wsp* locus and 34 of these also successfully amplified at the 16S rDNA locus. Sequence analysis identified five different *wsp* alleles of supergroup A. Two *wsp* alleles (661 and 11) were found to co-occur in individuals of six species (*B. bryoniae*, *B. decurtans*, *B. frauenfeldi, B. neohumeralis*, *B. strigifinis* and *D. axanus*); beyond this, *wsp* 661 occurred as a single sequence in one *B. frauenfeldi*, four *B. tryoni* and three *B. neohumeralis* individuals; and *wsp* 11 in one *B. neohumeralis* individual (Table 3, Fig. 2). *Wolbachia* MLST characterisation linked *wsp* 661 and *wsp* 11 with strains ST-285 and ST-289 respectively, demonstrating a high degree of sharing of *Wolbachia* strains among six tephritid species [28]. PCR-RFLP and sequencing confirmed the absence of any other detectable *wsp* variants in these individuals.

**Table 3:** *wsp* allele numbers and GenBank accession numbers of analysed individuals

Species (double/single infection)	Individual ID No.	16SrRNA	*wsp*†	*wsp* GenBank accession no.	MLST ST [28]
*B. bryoniae* (d)	157, 536, 545	yes	11	KC668327	289
			**661**	KC668326	285
*B. decurtans* (d)	85	yes	11	KC668325	289
			**661**	KC668324	285
*B. frauenfeldi* (d)	136	yes	16	KC668321	17
			^a^	KC693012	370
*B. frauenfeldi* (d)	485, 492	yes	11		289
			**661**		285
*B. frauenfeldi* (s)	490	yes	**661**		285
*B. neohumeralis* (d)	35, 109, 160, 221, 238, 248, 342, 345, 355	yes	11	KC668323	289
			**661**	KC668320	285
*B. neohumeralis* (s)	244	yes	11	KC668323	289
*B. neohumeralis* (s)	240, 243, 346	yes	**661**	KC668320	285
*B. strigifinis* (d)	81, 269, 503, 504	yes	11	KC668329	289
			**661**	KC668328	285
*B. tryoni* (s)	275, 276	yes	**661**	KC668332	285
*D. axanus* (d)	88	yes	11	KC668331	289
			**661**	KC668330	285
*B. perkinsi* (pseudogene)	74	yes	**662**	KC668319	n.a.

Amplicons were sequenced and confirmed by PCR-RFLP (Additional file 1); †*wsp* allele numbers from *Wolbachia wsp* database, new *wsp* alleles in bold; ^a^ *wsp* allele number not assigned

Cloning of *wsp* from one *B. frauenfeldi* individual (ID136) revealed two alleles unlike those found in the other *B. frauenfeldi* individuals: one sequence identical to *wsp* 16 of *Drosophila simulans* strain *w*Ri [53]; the other [GenBank: KC693012] with high similarity to two *wsp* sequences detected in *B. dorsalis* from China [22]. Furthermore, the *wsp* and MLST alleles were shared with the *Wolbachia* infections found in the parasitoid *F. arisanus* [28].

All five *B. perkinsi* individuals produced *wsp* and 16S rDNA amplicons. Cloning and sequencing of the *wsp* fragments revealed a novel allele, *wsp* 662, as well as multiple clones of a *wsp* sequence with a single base insertion. This insertion disrupts the open reading frame by incorporating stop codons, and may represent a *Wolbachia* pseudogene transferred to the host genome of this species. Amplification of the *ftsZ* locus in all individuals of *B. perkinsi* have previously failed during the MLST characterisation of *Wolbachia* in this species while all other loci were positive with A group like sequences [28]. We interpreted this as presence of multiple pseudogenes and absence of true *Wolbachia* infections in the analysed specimens of this species.

For *B. peninsularis*, the 16S rDNA fragment was consistently amplified from all seven individuals, whereas the *wsp* locus failed to amplify. Direct sequencing of the 16S rDNA amplicon showed multiple peaks in the sequence chromatogram indicating two distinct sequences: one full length B group 16S rDNA sequence, and one sequence with a 31 bp deletion at positions 182–213, indicative of a potential pseudogene. The sequence of the *B. peninsularis* full-length 16S rDNA fragment was deposited in GenBank, accession number KC775793. Failure to detect a *wsp* sequence in any of these individuals, and prior failure to detect the five MLST markers except *fbpA* (new allele 196) [28], suggested that this sequence may not represent a genuine *Wolbachia* infection; consequently this species was classified as uninfected. As a control for the 16S rDNA PCR assays, one *B. neohumeralis* sample, ID248, was chosen for 16S rDNA sequencing to confirm homology to other *Wolbachia* 16S rDNA sequences in GenBank (KC775794 and KC775795).

### Analysis of *Wolbachia* incidence and prevalence

Geographically as well as irrespective of host species and *Wolbachia* strains, *Wolbachia* was detected in five of 131 samples from equatorial Queensland (3.8 %), 22 of 195 (11.3 %) samples from tropical Queensland and the Northern Territory, and 6 of 179 (3.4 %) samples from subtropical Queensland. However, none of the 60 samples from temperate New South Wales and none of the 27 samples from central Australia had detectable *Wolbachia* (Additional file 1). The analysis of *Wolbachia* incidence within species revealed that it was restricted to the northern half of Australia (Fig. 1); analysis of species for which at least ten individuals per species were available for each half revealed *Wolbachia* incidence in at least one specimen for a number of these species in the northern half of the gradient while none was infected in the southern half. Therefore, incidence of *Wolbachia* was higher in the northern half of the gradient, with *Wolbachia* incidence in seven out of nine species for which a minimum of ten individuals were tested; all four species from the southern half for which a minimum of ten individuals per species were available in that region, were negative (Fisher’s exact test; *p* = 0.021). A linear model was applied in order to appropriately represent both widespread and more tropically restricted species and this detected a subtle relationship between the prevalence of *Wolbachia* and the midpoint latitude of twelve individual species (R^2^ = 0.86, F_1,10_ = 71.43; *p* < 0.001; Additional file 4).

For the seven species that had individuals with a full *Wolbachia* MLST profile plus *wsp*, the prevalence ranged from 2.1 % (4/190) to 16.7 % (1/6; Table 1). Multivariate analysis of infection prevalence in the only two widespread species that are endemic from equatorial to temperate climes, *B. neohumeralis* and *B. tryoni* (Table 2), detected a significant interaction of *Wolbachia* prevalence by latitude (Dev = 38.49, *p* < 0.05; Table 4) with *Wolbachia* being more common in the northern regions of east coast Australia. Univariate analysis with adjusted p-values (p.uni = adjusted) to account for family-wise error across both species did not detect a significant interaction (*p* > 0.05), demonstrating that the detectable effect on a single species is weak, but the pattern is strengthened when analysing both species together.

**Table 4:** Analyses of *Wolbachia* prevalence at different latitudes of *B. neohumeralis* and *B. tryoni*

Analysis of Deviance	Model: manyglm(formula = WolbPrevalence ~ latitude *status, family = "negative.binomial")			
Multivariate test:	Residual Df	Df	Deviance	Pr(>Dev)
intercept	39			
latitude	30	9	16.47	0.481
status	29	1	123.06	0.001***
latitude:status	20	9	38.49	0.042*
Univariate test:	*B. neohumeralis*		*B. tryoni*	
	Deviance	Pr(>Dev)	Deviance	Pr(>Dev)
latitude	12.14	0.35	4.33	0.81
status	48.57	0.001***	74.50	0.001***
latitude:status	25.73	0.128	12.76	0.128

Table presents Analysis of Deviance for multivariate and univariate analyses of *Wolbachia* prevalence (status) at different latitudes of two widespread species *B. neohumeralis* and *B. tryoni*, as per output of the mvabund package. *P*-value (Pr(>Dev)) Significance codes: 0 ‘***’ 0.001 ‘**’ 0.01 ‘*’ 0.05 ‘.’ 0.1 ‘’ 1

Furthermore, temporal effects on *Wolbachia* prevalence within *B. bryoniae, B. frauenfeldi*, *B. neohumeralis*, *B. strigifinis* and *B. tryoni* were tested for equatorial and tropical samples by using a Fisher’s exact test (α = 0.05) over the sampling years. No significant temporal change within the tested regions was detected, except for *B. tryoni* (*p* < 0.05), but this represented neither an overall increase nor decrease in prevalence over time (Additional file 5).

### Analysis of mitochondrial DNA

**Fig. 3:**
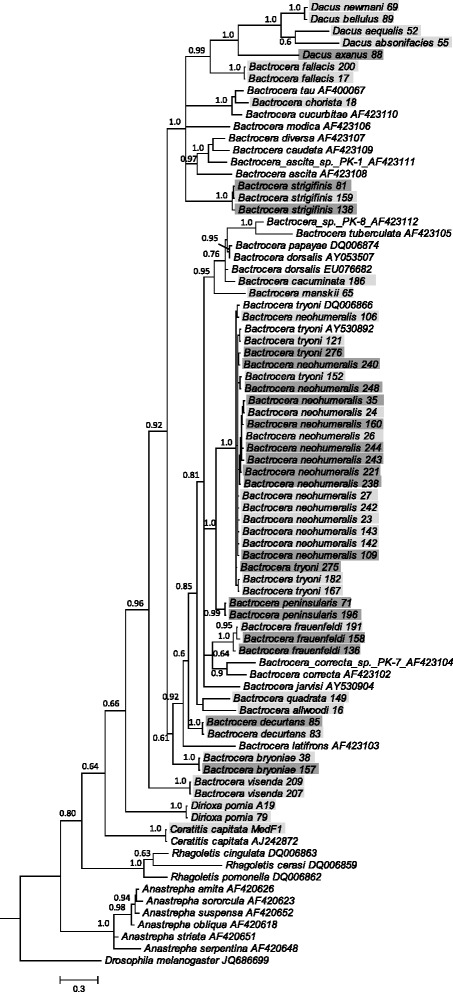
Bayesian inference tree of *COI* sequences from tephritid fruit flies. Representatives of the five major tephritid fruit fly genera, *Anastrepha, Bactrocera*, *Ceratitis*, *Dacus* and *Rhagoletis* as well as *Dirioxa* are shown. Dark grey shading highlights the individuals from this study harbouring *Wolbachia*, light grey highlights specimens that were negative for *Wolbachia*. Sequences from species without shading were retrieved from GenBank (accession numbers shown. Note: name for *B. dorsalis* has been retained as *B. papayae* DQ006874 as per GenBank entry, although these species names have been synonymised [40]). Tree was rooted with *Drosophila melanogaster*, and nodes are labelled with posterior probability values. Scale bar represents the number of nucleotide substitutions per site

Most tephritid species produced clear *COI* sequences. *Bactrocera jarvisi*, *B. murrayi*, *B. dorsalis* and *B. perkinsi* individuals produced ambiguous sequences, indicative of potential nuclear mitochondrial (numt) DNA in these species. The latter sequences were not included in phylogenetic analysis. Instead, *B. jarvisi* and *B. dorsalis* sequences were retrieved from GenBank. Bayesian analysis of 81 sequences (52 sequences from this study) over 571 bp returned a well-supported consensus tree (Fig. 3).

Mitochondrial haplotypes of morphologically identified individuals of different species harbouring identical *wsp* alleles were different, except for the two sibling species *B. neohumeralis* and *B. tryoni*; both had individuals with identical *Wolbachia* and mitochondrial sequences but without any linkage between *Wolbachia* infection and haplotypes. *COI* distance measures of *B. neohumeralis* and *B. tryoni* (analysed as one species complex) showed little difference within infected (1.22 %) and uninfected individuals (1.36 %), and between infected and uninfected individuals (1.31 %; Additional file 6; Fig. 3).

## Discussion

We surveyed 24 Australian tephritid fruit fly species throughout their natural range, and detected *Wolbachia* sequences in individuals of nine species. In two species, all individuals had pseudogenes and incomplete sets of marker genes – indicative of potential lateral gene transfer to the host genome without the presence of an existing infection. Both species are restricted to the tropics. The remaining seven fruit fly species had both 16S rDNA and *wsp* sequences characteristic for four *Wolbachia* strains for which complete MLST profiles had previously been established [28]. The four *Wolbachia* strains occurred as paired or single infections in more than one host species, indicative of horizontal *Wolbachia* transmission events into these species. Overall the incidence of *Wolbachia* strains (mostly two strains, ST-285 and ST-289) was restricted to tropical regions, and the prevalence of the four *Wolbachia* strains was very low in infected species (2-17 %). In five infected species (with enough samples available to test for a time effect), *Wolbachia* prevalence appeared to not have changed over time. In *B. neohumeralis* and *B. tryoni*, *Wolbachia* was not linked with any particular mitochondrial haplotypes. We thus concluded that the detected strains may not necessarily manipulate reproduction of their host species and maternal inheritance may also be unreliable. Therefore, the detection of identical *Wolbachia* strains at low prevalence in several species of this fruit fly community may be due to a spillover from a yet unknown source species, without the propagation of *Wolbachia* in any of the analysed host species, and this spillover appears to be restricted to the tropical regions.

### *Wolbachia* strains and pseudogenes

The previous MLST characterisation in Australian tropical tephritids revealed four complete MLST profiles of supergroup A *Wolbachia* in seven species [28]. Here, however, 16S rDNA and *wsp* sequences were isolated from nine and eight species, respectively while complete marker gene sets were not found for all nine species, which is likely to be due to lateral gene transfer of *Wolbachia* into the host genome*.* More specifically, all individuals of *B. perkinsi* produced 16S rDNA and *wsp* sequences (including some *wsp* sequences with stop codons). In the previous study, no individuals of this species amplified *ftsZ* and produced both pseudogenic and novel ORF sequences for other MLST loci [28]. Another species, *B. peninsularis,* also had signs of *Wolbachia* lateral gene transfer, as all individuals only amplified 16S rDNA but not *wsp* in this study while previously *fbpA* was detected as the only positive MLST locus in all individuals of this species [28]. Similarly, pseudogenised 16S rDNA and *fbpA* sequences were found in one out of eight tested *Glossina* fly species [15]. We thus conclude that the *Wolbachia* signals in *B. peninsularis* and *B. perkinsi* are due to lateral gene transfer from *Wolbachia* into these host genomes, as has been reported for other insects [14]. It may also be noted that the *Wolbachia* pseudogenic sequences were different enough to conclude that they did not derive from the four strains detected in this study.

Two of the previously characterised *Wolbachia* strains ST-285 and ST-289 [28] were found with their 16S rDNA and *wsp* sequences in individuals of six fruit fly species. Single infections by both of these strains were detected in *B. neohumeralis* individuals and single infections by ST-285 in all four infected *B. tryoni* individuals and one *B. frauenfeldi* individual. Two additional strains, ST-17 and ST-370, previously characterised by Morrow *et al.* [28] were detected with their 16S rDNA and *wsp* sequence variants in another *B. frauenfeldi* individual. It was demonstrated that this double infection is identical to *Wolbachia* from the fruit fly parasitoid *F. arisanus*, and this could be a spillover from this parasitoid species. This conclusion can be drawn based on the result of our earlier study in which all individuals of this parasitoid species were infected, including individuals from the Sydney region where fruit fly host populations were uninfected [28]. Of additional interest is that both *F. arisanus* and *B. frauenfeldi* are not endemic to Australia; *F. arisanus* was introduced to Australia in 1956 and now occurs from northern Queensland to Sydney [36, 54,55] while *B. frauenfeldi* was first detected on Cape York Peninsula in 1974 from where it has spread throughout northern Queensland [55]. This also means that ST-17 and ST-370 were only found in two host species that were originally not native to Australia.

### Latitudinal distribution of *Wolbachia* in Australian tephritids

After excluding pseudogenes in two species as potential *Wolbachia* infections, we detected *Wolbachia* in 29 % of tested Australian tephritid species, and this is lower than previous estimates of 40 % incidence of infections in arthropods, mostly based on a European dataset [1, 48]. Our results varied between climatic regions. *Wolbachia* incidence was restricted to equatorial and tropical regions north of Gladstone, while *Wolbachia* was absent from individuals caught in the more southern subtropical, temperate and arid inland regions. Specifically, *Wolbachia* was found in species whose range is limited to tropical and equatorial Queensland (*B. decurtans*, *B. frauenfeldi, B. strigifinis*); in species with a broader climatic range, *Wolbachia* occurred as far south as Cairns for *B. bryoniae*, Mackay for *B. tryoni* and Gladstone for *B. neohumeralis*, but was absent from individuals in more southerly subtropical and temperate regions. Within all of these species, *Wolbachia* prevalence was very low (2–17 %), and in the more widely distributed species *B. neohumeralis* and *B. tryoni*, *Wolbachia* was also restricted to the northern ranges of each species.

### *Wolbachia* in Australian tephritids: transient infections resulting from a spillover?

Previously, identical *Wolbachia* strains were found in multiple species of fruit flies that share ecological niches, but are unlikely to hybridise in nature, except possibly for *B. tryoni* and *B. neohumeralis* [28]. This study found that only low frequency *Wolbachia* signals were detected in samples from tropical environments, including for those species that are widespread from tropical to temperate climes. Furthermore, no *Wolbachia* was detected in any laboratory colonies of these species, including those originating from tropical locations, a result that was supported by 454 pyrosequencing of 16S rDNA amplicon libraries from these same individuals [41]. This suggests that there is ample opportunity for the horizontal transfer of *Wolbachia*, but the low prevalence, and lack of increasing prevalence over time, does not unequivocally show that these infections confer any selective advantage to infected females e.g. through reproductive manipulation such as cytoplasmic incompatibility (CI) nor that they are being reliably inherited. Experimental evidence of a heritable *Wolbachia* infection is necessary to confirm an established infection. In *Drosophila suzukii*, a similarly low frequency *Wolbachia* infection occurs with variable maternal transmission; however no CI or fitness benefits (such as increased fecundity, starvation resistance or desiccation resistance) were observed, and maintenance of *Wolbachia* in this species has yet to be explained [56]. Here, in the absence of any evidence of vertical inheritance, the finding of two strains (ST-285 and ST-289) in six host species at low prevalence is difficult to explain, and perhaps more likely due to a spillover of *Wolbachia* from an as yet unknown host species without any proliferation in any of the studied species. This interpretation still lacks direct experimental evidence, but transient infections are frequently observed in *Wolbachia* microinjection studies that detect *Wolbachia* in many individuals emerging from microinjected embryos. However, only a few injected embryos result in the establishment of stably inherited matrilines while *Wolbachia* is lost from most other matrilines [11], or remains undetectable in some matrilines for many generations [57] . In our study, the potential spillover of *Wolbachia* is restricted to the tropical regions, and is perhaps due to the higher biodiversity, resulting in increased ecological interactions in the tropics. The finding of two strains (ST-17 and ST-370) that are shared between fruit fly and parasitoid individuals supports the interpretation of a potential spillover with the parasitoid being the source in this instance; ST-17 and ST-370 were only found in one *B. frauenfeldi* individual while all *F. arisanus* individuals were infected with both strains, including from regions where host fruit flies were uninfected [28]. If, however, the infections are heritable in the studied fruit fly species, then the latitudinal restriction of *Wolbachia* incidence and prevalence in Australian fruit flies to northern Australia may be due to (1) *Wolbachia* loss due to environmental conditions of their hosts in the southern range or (2) an ongoing *Wolbachia* invasion of fruit fly species from north to south, driven by a reproductive phenotype.

An increasing number of studies have demonstrated that *Wolbachia* responds to the climatic environment of its host insects. For example, precipitation frequency appeared to correlate with the distribution of single and double infections in leaf beetle individuals in Panama, with multiple infections restricted to wetter regions [58]. In another study, multiple infections were found to be more frequent in one tropical habitat when compared with two temperate habitats [20]. Temperature is known to affect *Wolbachia* titres in a number of insect hosts. For example, the virulent *w*MelPop strain appears to over-replicate at elevated temperatures [59]. However, high temperatures may also effectively reduce *Wolbachia* densities in hosts through increased *Wolbachia*-bacteriophage activity [60], and thus result in diminished penetrance of CI or male-killing phenotypes [61, 62]. It is possible that temperature fluctuations and extremes, commonly found in southern regions of Australia, may be selecting more strongly against *Wolbachia* than the more constant temperature conditions in the tropics. Such climatic effects could also be contributing to the distribution of *Wolbachia* in previously reported analyses such as *D. melanogaster* [26] and cat fleas [63]. Similarly, climate and latitude were found to determine ranges of other microbial symbioses, for example in marine invertebrates [64], terrestrial insects [65–67] and humans [68].

The second scenario supposes that the *Wolbachia* in the Australian tephritid species, in particular infections of the widespread *B. tryoni* and *B. neohumeralis*, is relatively recently acquired, and a progressive CI driven sweep may therefore be in its infancy. However this hypothesis is less likely for the following reasons; a CI driven invasion should result in an increase of infection prevalence over time that was not detected in these species. Furthermore, *Wolbachia*-induced CI is expected to cause a selective sweep of infected mitochondrial haplotypes [69], yet there does not appear to be support for this in the *B. tryoni* and *B. neohumeralis* species complex [38] because both species share mitochondrial haplotypes across infected and uninfected individuals. We were not able to directly assess the CI phenotype of *Wolbachia* in Australian tephritids due to the lack of infected laboratory populations. The CI characterisation of the *Wolbachia* strains will thus require future field collection efforts, in particular of females, in order to set up infected laboratory colonies for crossing experiments. Testing of field females will also avoid a potential underestimate of *Wolbachia* infection rates due to our male-biased sampling approach that would not detect male-killing *Wolbachia* strains. However, prevalence of male-killing *Wolbachia* may be generally low as found for another fruit fly family, Drosophilidae [70].

### Evidence for numtDNA in *Bactrocera*

Blacket *et al.* [71] have previously detected numtDNA amplicons for *B. tryoni* and *B. neohumeralis* by using primers for a different section of *COI*; our different primer set did not yield numtDNA for *B. tryoni* and *B. neohumeralis* but revealed potential numtDNA sequences in other species such as *B. jarvisi*, *B. dorsalis, B. murrayi* and *B. perkinsi*. Interestingly, potential transfer of both mitochondrial and *Wolbachia* genes into the nuclear host genome were detected for *B. perkinsi*.

## Conclusions

In this study of Australian tephritids we detected several *Wolbachia* pseudogenes that may be host-genomic footprints of historic infections that have since been lost from host species. Within the species of relevance (*B. peninsularis* and *B. perkinsi*), these pseudogenes were fixed at 100 % while prevalence of *Wolbachia* infections in other species was overly low (<20 %). Under the assumption that the detected *Wolbachia* strains in seven fruit fly species may not be reliably inherited nor cause a reproductive phenotype, these infections could be considered transient and a result of a spillover from a yet unknown source (while we have identified a potential *Wolbachia* spillover from *F. arisanus* to *B. frauenfeldi*). In our study system, this spillover appears to be restricted to northern Australia and has not resulted in further proliferation within any of the tested host lineages, and this warrants future investigation. The fact that we have not found a single case of a high prevalence infected Australian tephritid fruit fly species in 24 tested species raises the question whether these taxa exhibit traits that reduce *Wolbachia* infectivity while two species had *Wolbachia* infections in their evolutionary past. Other related pest taxa such as olive fly *Bactrocera oleae* appear uninfected in natural populations while laboratory populations were successfully infected [72]; a high incidence of *wsp* genes was found in Thai *Bactrocera* species [73, 74] while prevalence of *wsp* in *B. dorsalis* was found to be extremely low [21], although these strains were not further characterised. Perhaps some of these *Bactrocera* species also contain *Wolbachia* signals that are either pseudogenes and/or transient somatic infections, and similar questions may be valid for *Wolbachia* signals found in other host species. Our study highlights that tropical insect communities have signs of lateral gene transfer and may be exposed to increased horizontal *Wolbachia* transfer. It also emphasises that global estimates for *Wolbachia* infection frequencies should be interpreted with caution, and account for lateral gene transfer, variable prevalence within species and transient spillover effects. This may be of particular relevance if *Wolbachia* surveys are based on a single or a few marker genes for detection of infections in a host species, and without a broader sampling effort from the host’s distribution (also see [75]).

## Availability of supporting data

Data sets supporting the results of this article are included within the article and its additional files. DNA sequences generated in this study were submitted to GenBank: accession numbers KC581371–KC581411, KC693011, KC775785-KC775792 (*COI*) and KC668319-KC668321, KC668323-KC668332, KC693012 (*wsp*) and KC775793-KC775795 (16S rDNA). New *wsp* alleles have also been deposited in the *Wolbachia wsp* database (http://pubmlst.org/wolbachia/wsp/).

## Additional files

Additional file 1:
**Collection data for field-caught fruit flies from this study.** Information for collection location, climate group and year, specimen specific ID number, sex, result for *wsp* and 16S rDNA screening and GenBank accession number of *COI* partial gene are included. (CSV 61 kb) Additional file 2:
**Laboratory strains screened for** ***Wolbachia*** **using** ***wsp*** **and 16S rDNA.** (PDF 104 kb) Additional file 3:
**PCR conditions and primer sequences used in this research.** (PDF 103 kb) Additional file 4:
**Relationship between prevalence of** ***Wolbachia*** **infection in twelve tephritid species and the midpoint of their latitudinal distribution, based on Hancock** ***et al.*** **[** 32 **] and Royer and Hancock [** 33 **] (R** ^**2**^ **= 0.86, F**_**1,10**_ **= 71.43;** ***p*** **< 0.001).** At least ten individuals were tested for uninfected species (*B. cacuminata, B. chorista, B. jarvisi, D. aequalis, D. pornia*). (PDF 126 kb) Additional file 5:
**Comparison of** ***Wolbachia*** **infected individuals sampled from equatorial and tropical regions for (A)** ***B. bryoniae*** **, (B)** ***B. frauenfeldi*** **, (C)** ***B. neohumeralis,*** **(D),** ***B. strigifinis*** **and (E)** ***B. tryoni*** **over five collection years.** Fisher’s exact test shows overall no significant (ns) temporal effect at α = 0.05, except for *B. tryoni,* but this did not represent an overall increase or decrease in prevalence. (PDF 100 kb) Additional file 6:
**Distance matrix of** ***COI*** **sequences covering 551 bp positions of** ***B. tryoni*** **and** ***B. neohumeralis*** **individuals, grouped by** ***Wolbachia*** **-infected (grey shading) and uninfected (no shading).** Below the diagonal are the pairwise number of differences and above the diagonal are p-distance values. P-distances given for within- and between- group means are listed below the main table. (PDF 103 kb)
