# Supplementary material for: Wolbachia pseudogenes and low prevalence infections in tropical but not temperate Australian tephritid fruit flies: manifestations of lateral gene transfer and endosymbiont spillover?
Source: BMC Evol Biol. 2015 Sep 18;15:202. doi: 10.1186/s12862-015-0474-2 (PMC4575488; doi:10.1186/s12862-015-0474-2)
Supplement: Additional file 2: — Laboratory strains screened for Wolbachia using wsp and 16S rDNA. (PDF 104 kb) [file 12862_2015_474_MOESM2_ESM.pdf]

Additional File 2. Laboratory strains screened for *Wolbachia* using *wsp* and 16S rDNA.

Species	Stock line	Laboratory location*	Sampling year	Individual name	16S	*wsp*	COI accession no.
Bactrocera neohumeralis	Bneo UWS	Richmond, UWS	2012	Bn1	n	n	
Bactrocera neohumeralis	Bneo UWS	Richmond, UWS	2012	Bn2	n	n	
Bactrocera neohumeralis	Bneo UWS	Richmond, UWS	2012	Bn3	n	n	
Bactrocera neohumeralis	Bneo UWS	Richmond, UWS	2012	Bn4	n	n	
Bactrocera neohumeralis	Bneo UWS	Richmond, UWS	2012	Bn5	n	n	
Bactrocera neohumeralis	Bneo UWS	Richmond, UWS	2012	Bn6	n	n	
Bactrocera neohumeralis	Bneo UWS	Richmond, UWS	2012	Bn7	n	n	
Bactrocera neohumeralis	Bneo UWS	Richmond, UWS	2012	Bn8	n	n	
Bactrocera cacuminata	Bcac UWS	Richmond, UWS	2012	Bcac1	n	n	KC581374
Bactrocera cacuminata	Bcac UWS	Richmond, UWS	2012	Bcac2	n	n	KC581374
Bactrocera cacuminata	Bcac UWS	Richmond, UWS	2012	Bcac3	n	n	
Bactrocera cacuminata	Bcac UWS	Richmond, UWS	2012	Bcac4	n	n	
Bactrocera cacuminata	Bcac UWS	Richmond, UWS	2012	Bcac5	n	n	
Bactrocera cacuminata	Bcac UWS	Richmond, UWS	2012	Bcac6	n	n	
Bactrocera cacuminata	Bcac UWS	Richmond, UWS	2012	Bcac7	n	n	
Bactrocera cacuminata	Bcac UWS	Richmond, UWS	2012	Bcac8	n	n	
Bactrocera tryoni	BtHAC	Richmond, UWS	2012	HAC1	n	n	
Bactrocera tryoni	BtHAC	Richmond, UWS	2012	HAC2	n	n	
Bactrocera tryoni	BtHAC	Richmond, UWS	2012	HAC3	n	n	
Bactrocera tryoni	BtHAC	Richmond, UWS	2012	HAC4	n	n	
Bactrocera tryoni	BtHAC	Richmond, UWS	2012	HAC5	n	n	
Bactrocera tryoni	BtHAC	Richmond, UWS	2012	HAC6	n	n	
Bactrocera tryoni	BtHAC	Richmond, UWS	2012	HAC7	n	n	
Bactrocera tryoni	BtHAC	Richmond, UWS	2012	HAC8	n	n	
Bactrocera tryoni	BtGOS	Richmond, UWS	2012	GOS1	n	n	
Bactrocera tryoni	BtGOS	Richmond, UWS	2012	GOS2	n	n	
Bactrocera tryoni	BtGOS	Richmond, UWS	2012	GOS3	n	n	
Bactrocera tryoni	BtGOS	Richmond, UWS	2012	GOS4	n	n	
Bactrocera tryoni	BtGOS	Richmond, UWS	2012	GOS5	n	n	
Bactrocera tryoni	BtGOS	Richmond, UWS	2012	GOS6	n	n	
Bactrocera tryoni	BtGOS	Richmond, UWS	2012	GOS7	n	n	
Bactrocera tryoni	BtGOS	Richmond, UWS	2012	GOS8	n	n	
Bactrocera jarvisi	Bjar UWS	Richmond, UWS	2012	Bj1	n	n	
Bactrocera jarvisi	Bjar UWS	Richmond, UWS	2012	Bj2	n	n	
Bactrocera jarvisi	Bjar UWS	Richmond, UWS	2012	Bj3	n	n	
Bactrocera jarvisi	Bjar UWS	Richmond, UWS	2012	Bj4	n	n	
Bactrocera jarvisi	Bjar UWS	Richmond, UWS	2012	Bj5	n	n	
Bactrocera jarvisi	Bjar UWS	Richmond, UWS	2012	Bj6	n	n	
Bactrocera jarvisi	Bjar UWS	Richmond, UWS	2012	Bj7	n	n	
Bactrocera jarvisi	Bjar UWS	Richmond, UWS	2012	Bj8	n	n	

Species	Stock line	Laboratory location*	Sampling year	Individual name	16S	*wsp*	COI accession no.
Bactrocera neohumeralis	Bneo09	Cairns, QDAF	2012	Bn09.1	n	n	
Bactrocera neohumeralis	Bneo09	Cairns, QDAF	2012	Bn09.2	n	n	
Bactrocera neohumeralis	Bneo09	Cairns, QDAF	2012	Bn09.3	n	n	
Bactrocera neohumeralis	Bneo09	Cairns, QDAF	2012	Bn09.4	n	n	
Bactrocera neohumeralis	Bneo09	Cairns, QDAF	2012	Bn09.5	n	n	
Bactrocera neohumeralis	Bneo09	Cairns, QDAF	2012	Bn09.6	n	n	
Bactrocera neohumeralis	Bneo09	Cairns, QDAF	2012	Bn09.7	n	n	
Bactrocera neohumeralis	Bneo09	Cairns, QDAF	2012	Bn09.8	n	n	
Bactrocera neohumeralis	Bneo12	Cairns, QDAF	2012	Bn12.1	n	n	
Bactrocera neohumeralis	Bneo12	Cairns, QDAF	2012	Bn12.2	n	n	
Bactrocera neohumeralis	Bneo12	Cairns, QDAF	2012	Bn12.3	n	n	
Bactrocera neohumeralis	Bneo12	Cairns, QDAF	2012	Bn12.4	n	n	
Bactrocera neohumeralis	Bneo12	Cairns, QDAF	2012	Bn12.5	n	n	
Bactrocera neohumeralis	Bneo12	Cairns, QDAF	2012	Bn12.6	n	n	
Bactrocera neohumeralis	Bneo12	Cairns, QDAF	2012	Bn12.7	n	n	
Bactrocera neohumeralis	Bneo12	Cairns, QDAF	2012	Bn12.8	n	n	
Ceratitis capitata	MedFly	Perth, DAFWA	2011	MF1	n	n	KC581412
Ceratitis capitata	MedFly	Perth, DAFWA	2011	MF2	n	n	
Ceratitis capitata	MedFly	Perth, DAFWA	2011	MF3	n	n	
Ceratitis capitata	MedFly	Perth, DAFWA	2011	MF4	n	n	
Ceratitis capitata	MedFly	Perth, DAFWA	2011	MF5	n	n	
Ceratitis capitata	MedFly	Perth, DAFWA	2011	MF6	n	n	
Ceratitis capitata	MedFly	Perth, DAFWA	2011	MF7	n	n	
Ceratitis capitata	MedFly	Perth, DAFWA	2011	MF8	n	n	

*UWS (University of Western Sydney), QDAF (Queensland Department of Agriculture and Fisheries), DAFWA (Department of Agriculture and Food, Western Australia)
