# Supplementary material for: Wolbachia pseudogenes and low prevalence infections in tropical but not temperate Australian tephritid fruit flies: manifestations of lateral gene transfer and endosymbiont spillover?
Source: BMC Evol Biol. 2015 Sep 18;15:202. doi: 10.1186/s12862-015-0474-2 (PMC4575488; doi:10.1186/s12862-015-0474-2)
Supplement: Additional file 3: — PCR conditions and primer sequences used in this research. (PDF 103 kb) [file 12862_2015_474_MOESM3_ESM.pdf]

Additional File 3. PCR conditions and primer sequences used in this research

	wsp	*16S*	*COI* and Sequencing	DIG-labelled *wsp* probe
**PCR protocol**				
Total volume	10µL / 20µL	10µL	20µL	50µL
GoTaq Reaction Buffer	1X	1X	1X	1X
$MgCl_2$	4mM	2mM	2.5mM	5mM
dNTP	150µM each	125µM each	200µM each	2.5µL (DIG-dNTP labelling mixture)
Primer 1	0.8µM	0.63µM	0.4µM	0.6µM
Primer 2	0.8µM	0.63µM	0.4µM	0.6µM
GoTaq DNA polymerase	0.4u / 0.8u	0.5u	0.5u	1u
DNA Template	1µL / 2µL	1µL	2µL	2µL
**Thermal Cycling**				
Denaturation	94°C for 2min<br>94°C for 10s, 65°C for 30s, 68°C for 1min (x10 cycles)<br>94°C for 10s, 65°C for 30s, 68°C for 1min (x25 cycles, including an incremental increase of 20s at 68°C)	94°C for 5min<br>94°C for 30s, 54°C for 30s, 72°C for 1min (x35 cycles)<br>72°C for 10min	94°C for 5min<br>94°C for 30s, 55°C for 30s, 72°C for 1min (x35 cycles)<br>72°C for 10min	94°C for 2min<br>94°C for 10s, 65°C for 30s, 68°C for 1min (x10 cycles)<br>94°C for 10s, 65°C for 30s, 68°C for 1min (x25 cycles, including an incremental increase of 20s at 68°C)
**Primers**				
wsp				
Wsp-F	TGGTCCAATAAGTGATGAAGAAACTAGCTA	Jeyaprakash and Hoy, 2000		
Wsp-R	AAAAATTAAACGCTACTCCAGCTTCTGCAC	Jeyaprakash and Hoy, 2000		
16S				
wspecF	CATACCTATTCGAAGGGATAG	Werren and Windsor, 2000		
wspecR	AGCTTCGAGTGAAACCAATTC	Werren and Windsor, 2000		
COI				
Dick (C1-J-2441)	CCTACAGGAATTAAAATTTTTAGATGATTA	Simon et al. 1994		
Pat (TL2-N-3014)	TCCATTGCACTAATCTGCCATATTA	Simon et al. 1994		
Sequencing				
T7 Promoter	TAATACGACTCACTATAGGG			
SP6	ATTTAGGTGACACTATAG			
