# Supplementary material for: Wolbachia pseudogenes and low prevalence infections in tropical but not temperate Australian tephritid fruit flies: manifestations of lateral gene transfer and endosymbiont spillover?
Source: BMC Evol Biol. 2015 Sep 18;15:202. doi: 10.1186/s12862-015-0474-2 (PMC4575488; doi:10.1186/s12862-015-0474-2)
Supplement: Additional file 4: — Relationship between prevalence of Wolbachia infection in twelve tephritid species and the midpoint of their latitudinal distribution, based on Hancock et al. [ 32 ] and Royer and Hancock [ 33 ] (R 2 = 0.86, F 1,10 = 71.43; p < 0.001). At least ten individuals were tested for uninfected species (B. cacuminata, B. chorista, B. jarvisi, D. aequalis, D. pornia). (PDF 126 kb) [file 12862_2015_474_MOESM4_ESM.pdf]

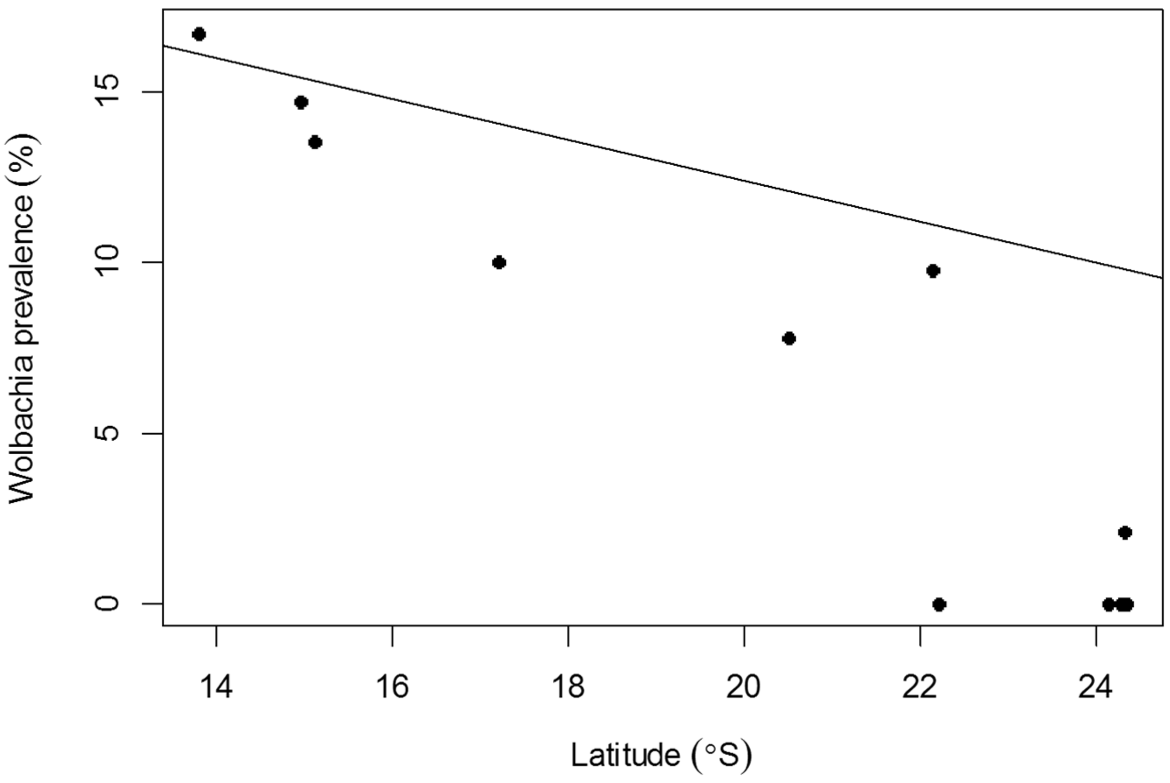

Additional File 4. Relationship between prevalence of *Wolbachia* infection in twelve tephritid species and the midpoint of their latitudinal distribution, based on Hancock *et al*. [32] and Royer and Hancock [33] ($R^2 = 0.86$, $F_{1,10} = 71.43$; $p < 0.001$). At least ten individuals were tested for uninfected species (*B. cacuminata, B. chorista, B. jarvisi, D. aequalis, D. pornia*).
