# Supplementary material for: Wolbachia pseudogenes and low prevalence infections in tropical but not temperate Australian tephritid fruit flies: manifestations of lateral gene transfer and endosymbiont spillover?
Source: BMC Evol Biol. 2015 Sep 18;15:202. doi: 10.1186/s12862-015-0474-2 (PMC4575488; doi:10.1186/s12862-015-0474-2)
Supplement: Additional file 5: — Comparison of Wolbachia infected individuals sampled from equatorial and tropical regions for (A) B. bryoniae , (B) B. frauenfeldi , (C) B. neohumeralis, (D), B. strigifinis and (E) B. tryoni over five collection years. Fisher’s exact test shows overall no significant (ns) temporal effect at α = 0.05, except for B. tryoni, but this did not represent an overall increase or decrease in prevalence. (PDF 100 kb) [file 12862_2015_474_MOESM5_ESM.pdf]

Additional File 5. Comparison of *Wolbachia* infected individuals sampled from equatorial and tropical regions for (A) *B. bryoniae* (B), *B. frauenfeldi* (C) *B. neohumeralis,* (D) *B. strigifinis* and (E) *B. tryoni* over five collection years. Fisher's exact test shows overall no significant (ns) temporal effect at α = 0.05, except for *B. tryoni,* but this did not represent an overall increase or decrease in prevalence.

	**A**	**B**	**C**	**D**	**E**
Collection year	*B. bryoniae*	*B. frauenfeldi*	*B. neohumeralis*	*B. strigifinis*	*B. tryoni*
1997	0/7				
1998	1/8	0/3	2/14	3/12	0/23
2001	1/2	2/6	1/4	0/3	2/5
2012		0/5	10/64	0/4	
2013	2/28	3/20		2/18	2/65
p value	p = 0.222	p=0.612	p = 0.848	p = 0.610	p = 0.019*
