# Supplementary material for: Wolbachia pseudogenes and low prevalence infections in tropical but not temperate Australian tephritid fruit flies: manifestations of lateral gene transfer and endosymbiont spillover?
Source: BMC Evol Biol. 2015 Sep 18;15:202. doi: 10.1186/s12862-015-0474-2 (PMC4575488; doi:10.1186/s12862-015-0474-2)
Supplement: Additional file 6: — Distance matrix of COI sequences covering 551 bp positions of B. tryoni and B. neohumeralis individuals, grouped by Wolbachia -infected (grey shading) and uninfected (no shading). Below the diagonal are the pairwise number of differences and above the diagonal are p-distance values. P-distances given for within- and between- group means are listed below the main table. (PDF 103 kb) [file 12862_2015_474_MOESM6_ESM.pdf]

Additional File 6. Distance matrix of *COI* sequences covering 551bp positions of *Bactrocera tryoni* and *Bactrocera neohumeralis* individuals, grouped by *Wolbachia* -infected (grey shading) and uninfected (no shading). Below the diagonal are the pairwise number of differences and above the diagonal are p-distance values. P-distances given for within- and between- group means below the main table.

Species and individual ID. No.	*B. neohumeralis_106*	*B. neohumeralis_142*	*B. neohumeralis_143*	*B. neohumeralis_23*	*B. neohumeralis_24*	*B. neohumeralis_242*	*B. neohumeralis_26*	*B. neohumeralis_27*	*B. tryoni_121*	*B. tryoni_152*	*B. tryoni_167*	*B. tryoni_182*	*B. neohumeralis_109*	*B. neohumeralis_160*	*B. neohumeralis_221*	*B. neohumeralis_238*	*B. neohumeralis_240*	*B. neohumeralis_243*	*B. neohumeralis_244*	*B. neohumeralis_248*	*B. neohumeralis_35*	*B. tryoni_275*	*B. tryoni_276*
B. neohumeralis_106		0.016	0.016	0.018	0.027	0.016	0.022	0.016	0.011	0.020	0.015	0.016	0.016	0.025	0.020	0.018	0.018	0.022	0.025	0.016	0.025	0.009	0.016
B. neohumeralis_142	9		0.004	0.005	0.018	0.004	0.013	0.004	0.009	0.011	0.016	0.018	0.004	0.016	0.011	0.009	0.005	0.016	0.016	0.007	0.016	0.011	0.007
B. neohumeralis_143	9	2		0.005	0.018	0.004	0.013	0.004	0.009	0.011	0.016	0.018	0.004	0.016	0.011	0.009	0.005	0.016	0.016	0.007	0.016	0.011	0.007
B. neohumeralis_23	10	3	3		0.016	0.005	0.011	0.005	0.011	0.013	0.018	0.020	0.005	0.015	0.009	0.007	0.004	0.016	0.015	0.009	0.015	0.013	0.009
B. neohumeralis_24	15	10	10	9		0.018	0.005	0.015	0.020	0.018	0.024	0.025	0.018	0.005	0.007	0.009	0.016	0.011	0.009	0.018	0.002	0.022	0.022
B. neohumeralis_242	9	2	2	3	10		0.013	0.004	0.009	0.011	0.016	0.018	0.004	0.016	0.011	0.009	0.005	0.016	0.016	0.007	0.016	0.007	0.007
B. neohumeralis_26	12	7	7	6	3	7		0.009	0.015	0.013	0.018	0.020	0.013	0.004	0.002	0.004	0.011	0.005	0.004	0.013	0.007	0.016	0.016
B. neohumeralis_27	9	2	2	3	8	2	5		0.009	0.007	0.013	0.015	0.004	0.013	0.007	0.005	0.005	0.013	0.013	0.007	0.013	0.011	0.007
B. tryoni_121	6	5	5	6	11	5	8	5		0.013	0.018	0.020	0.009	0.018	0.013	0.011	0.011	0.018	0.018	0.009	0.018	0.013	0.013
B. tryoni_152	11	6	6	7	10	6	7	4	7		0.016	0.018	0.011	0.016	0.011	0.009	0.013	0.016	0.016	0.004	0.016	0.015	0.015
B. tryoni_167	8	9	9	10	13	9	10	7	10	9		0.002	0.016	0.022	0.016	0.015	0.018	0.022	0.022	0.016	0.022	0.009	0.020
B. tryoni_182	9	10	10	11	14	10	11	8	11	10	1		0.018	0.024	0.018	0.016	0.020	0.024	0.024	0.018	0.024	0.011	0.022
B. neohumeralis_109	9	2	2	3	10	2	7	2	5	6	9	10		0.013	0.011	0.009	0.005	0.016	0.016	0.007	0.016	0.011	0.007
B. neohumeralis_160	14	9	9	8	3	9	2	7	10	9	12	13	7		0.005	0.007	0.015	0.009	0.007	0.016	0.007	0.020	0.020
B. neohumeralis_221	11	6	6	5	4	6	1	4	7	6	9	10	6	3		0.002	0.009	0.007	0.005	0.011	0.009	0.015	0.015
B. neohumeralis_238	10	5	5	4	5	5	2	3	6	5	8	9	5	4	1		0.007	0.009	0.007	0.009	0.011	0.013	0.013
B. neohumeralis_240	10	3	3	2	9	3	6	3	6	7	10	11	3	8	5	4		0.016	0.015	0.009	0.015	0.013	0.005
B. neohumeralis_243	12	9	9	9	6	9	3	7	10	9	12	13	9	5	4	5	9		0.009	0.016	0.013	0.020	0.020
B. neohumeralis_244	14	9	9	8	5	9	2	7	10	9	12	13	9	4	3	4	8	5		0.016	0.011	0.020	0.020
B. neohumeralis_248	9	4	4	5	10	4	7	4	5	2	9	10	4	9	6	5	5	9	9		0.016	0.011	0.011
B. neohumeralis_35	14	9	9	8	1	9	4	7	10	9	12	13	9	4	5	6	8	7	6	9		0.020	0.020
B. tryoni_275	5	6	6	7	12	4	9	6	7	8	5	6	6	11	8	7	7	11	11	6	11		0.015
B. tryoni_276	9	4	4	5	12	4	9	4	7	8	11	12	4	11	8	7	3	11	11	6	11	8	

Group means	within group	between groups
Wolbachia negative	0.0136	0.0131
Wolbachia positive	0.0122	
